# Prevalence of anemia in diabetic adult outpatients in Northeast Ethiopia

**DOI:** 10.1371/journal.pone.0222111

**Published:** 2019-09-09

**Authors:** Temesgen Fiseha, Aderaw Adamu, Melkam Tesfaye, Angesom Gebreweld

**Affiliations:** Department of Clinical Laboratory Science, College of Medicine and Health Sciences, Wollo University, Dessie, Ethiopia; University of Oxford, UNITED KINGDOM

## Abstract

**Background:**

Anemia is a common finding in patients with diabetes, even in the absence of kidney disease and is a risk factor for adverse outcomes, including all-cause and cardiovascular mortality. Despite this, relatively little is known about the burden of anemia among adults with diabetes in sub-Saharan Africa. The aim of this study was to determine the prevalence of anemia and its association with renal disease among diabetic adult outpatients attending a hospital in Northeast Ethiopia.

**Methods:**

A cross-sectional study was conducted among 412 diabetic adults at the diabetes clinic of Dessie Referral hospital in Northeast Ethiopia, from January to April 2018. Each patient provided a blood sample for hemoglobin and serum creatinine levels and urine for albuminuria. Anemia was defined by World Health Organization criteria (<13 g/dl for men and <12 g/dl for women). Glomerular filtration rate (GFR) was estimated using the 4-variable Modification of Diet in Renal Disease (MDRD) equation. Chronic kidney disease (CKD) was classified into 5 stages based on the eGFR and albuminuria.

**Results:**

Anemia was present in 26.7% of the participants, and CKD in 43.0%. Anemia was more prevalent in patients with CKD (39.5%) than those without CKD (17.0%; *P <* 0.001). The prevalence of anemia increased with stage of CKD, from 22.6% at stage 1 to 100% at stage 4. Fifteen percent of the patients had anemia below the treatment threshold of 11 g ⁄dl. In multivariate analysis, older age (AOR = 2.41, 95% CI 1.11–5.21); type 2 diabetes (AOR = 2.40, 95% CI 1.14–5.08); presence of hypertension (AOR = 3.78, 95% CI 1.35–10.57); high systolic BP (AOR = 1.05, 95% CI 1.02–1.08); serum creatinine (AOR = 12.80, 95% CI 3.90–87.98) and low GFR (AOR = 9.50, 95% CI 4.05–22.28) were independently associated with greater odds for the presence of anemia

**Conclusions:**

Anemia is commonly present among diabetic adults attending our diabetes outpatient clinic in Northeast Ethiopia, including those without kidney disease. Our findings highlight the need for incorporating anemia screening into routine diabetes care to enable early detection and treatment of anemia and hence improve the overall care of patients with diabetes.

## Introduction

Anemia is a common and often unrecognized complication of diabetes associated with all-cause and cardiovascular disease mortality [1,2]. Anemia is known to contribute significantly to the development of micro- and macro-vascular complications of diabetes, which has a negative impact on the quality of life of the patients and healthcare costs of the disease [3–5]. Similarly, in patients with diabetes, anemia has been shown to be an independent risk factor for cardiovascular events and is associated with rapid decline of renal function and increased need for renal replacement therapy, which is often unavailable or unaffordable in most developing countries like Africa [6,7]. Early identification and treatment of anemia is, therefore, an important therapeutic strategy to reduce the risk of adverse outcomes and improve quality of life of patients with diabetes [8].

The risk of anemia in patients with diabetes is estimated to be two- to three fold higher than that of patients without diabetes [9,10]. Evidences indicate that the incidence and prevalence of anemia in patients with diabetes is typically associated with erythropoietin deficiency due to concomitant renal disease [11,12]. The risk of developing anemia in patients with diabetes and renal disease is significantly greater, and is often more severe and occurs earlier than in non-diabetic patients with renal diseases [13,14]. Recent studies have demonstrated the unfavorable influence of anemia on progression of renal disease, cost of managing the disease, quality of life and cardiovascular disease and all-cause mortality in diabetic patients who also have renal disease, and the combination of anemia and kidney disease in diabetics identifies a group with adverse outcomes [15–17]. There are, however, other factors that could contribute to the development of anemia in diabetes, including chronic inflammation, oxidative stress, autonomic neuropathy, nutritional deficiencies (iron, folate, and vitamin B_12_), autoimmune diseases, drugs and advanced glycation [11,18].

Despite the high prevalence of anemia in patients with diabetes, even in the absence of renal disease and its significant adverse effect on disease outcomes [10,19], very little research has been conducted on the burden of anemia among adults with diabetes in sub-Saharan Africa. This information will be useful in establishing the clinical relevance and need of incorporating anemia screening into the routine assessment of diabetic complications to improve diabetes care. Therefore, in this study we aimed to determine the prevalence of anemia and its association with renal disease among diabetic adult outpatients attending our clinic in Northeast Ethiopia.

## Methods and materials

### Study design, setting and population

We conducted a cross-sectional study at the out-patient diabetic clinic of Dessie Referral Hospital (DRH) in Northeast Ethiopia, from January to April 2018. DRH is found in Dessie town of Amhara regional state, which is located 401 km northeast of the capital Addis Ababa, Ethiopia, and it serves as a referral center for the Wollo and surrounding zones. The hospital registers and treats all diagnosed diabetic patients and provides tertiary diabetes patient care in the region. Patients with diabetes receive chronic care at the out-patient section of the diabetes clinic of the hospital. Patients with diabetes receiving care in the unit undergo an annual evaluation that includes amongst others: a clinical evaluation, an assessment of diabetes control (fasting blood glucose), chronic complications and cardiovascular risk factors assessment. Adult patients (≥ 18 years) attending the outpatient diabetes clinic of the hospital for routine follow up during the study period were included. All patients with a known hematologic disease, any acute condition within the last 2 weeks, or those who received a blood transfusion in the preceding 3 months were excluded. Patients were also excluded if they were pregnant (women) or hospitalized.

### Sample size determination

The required sample size of the study was determined using single population proportion formula by considering: 50% prevalence rate of anemia, Z = the level of statistical significance with a 95% confidence interval (CI) of 1.96, and a precision level of 0.05. Then the minimum sample size obtained was 384. After adding 10% to account for non-respondents, a total of 422 diabetic patients were included in the study. A systematic random sampling technique (i.e., every three patient) was employed to select the study participants. Ten patients with missing clinical data were excluded, leaving 412 patients for the final analysis.

### Data collection and laboratory measurements

Eligible patients were approached and invited to participate while they were attending the clinic for their regular follow-up. Consenting patients were interviewed by the study investigators to collect information on demographic (age, sex, residence and level of education), medical history (presence of hypertension/use of antihypertensive medication) and behavioral factors (smoking and alcohol consumption) using a structured questionnaire. Anthropometric data such as weight (kg), height (m), and blood pressure (mm Hg) also were collected by the attending nurse or doctor. The body mass index (BMI) was calculated as weight divided by the square of height (kg/m^2^). Blood pressure (BP) was taken using a manual sphygmomanometer in a sitting position, after 5 minutes of rest and three measurements were averaged to be recorded. Hypertension was defined as systolic BP ≥140 mmHg and/or diastolic BP ≥90 mmHg or a self-reported presence of hypertension/use of antihypertensive medication.

Clinical measures including duration of diabetes, type of diabetes and measurements of fasting blood glucose level were abstracted from patients’ database. Participants fasting blood glucose reading for at least three months were recorded for computing the mean blood glucose level. Blood samples were collected in the early morning after an overnight fasting and then analyzed at the hospital laboratory. Five ml venous blood was collected by vacutainer test tube coated EDTA anticoagulant for haemoglobin determination. Hemoglobin (Hgb) values were determined using the hematology analyzer Cell-Dyn 1800 (Abbott Laboratories Diagnostics Division, USA). Five ml of venous blood was obtained by using the serum separation tube (SST) and centrifuged within 1 hour for serum separation for creatinine measurement. Serum creatinine was measured using Jaffe kinetic method as mg/dl. Urine samples were also collected from the participating patients to detect albuminuria using dipsticks (COMBINA 11S, Human).

### Definitions

Anemia was defined according to the World Health Organization (WHO) criteria: Hgb concentration <13 g/dl for males and < 12 g/dl for females [20]. A second definition of anemia was also used based on the suggested threshold of Hgb < 11 g⁄dl (for both sexes) for the initiation of treatment with erythropoietin for anemia in chronic kidney disease (CKD) [21]. Glomerular filtration rate (GFR) was estimated by using the 4-variable the Modification of Diet in Renal Disease (MDRD) study equation [22]). Patients were classified into 5 stages of CKD based on eGFR and evidence of kidney damage, in accordance with the Kidney Disease Outcomes Quality Initiative (K/DOQI) guideline as: stage 1, an eGFR ≥ 90 ml/min/1.73 m^2^ with albuminuria; and stage 2, eGFR = 60–89.9 ml/min/1.73 m^2^ with albuminuria; and stages 3, 4 and 5 as an eGFR of 30–59.9, 15–29.9 and < 15 ml/min/1.73 m^2^, respectively. CKD was defined as K/DOQI CKD stages 1–5 (an eGFR of <60 ml/min/1.73 m^2^ and/or albuminuria) [23].

### Statistical analysis

The data was entered in to “EpiData version 3.1” and was exported to SPSS version 20.0 statistical software for analysis. Normally distributed and continuous variables were expressed as mean (± SD), and non-normally distributed variables were presented as medians (quartiles 25 and 75%). Chi squared (x^2^) test was used for comparison of categorical variables while the Student t-test (or in case of asymmetry the Kruskal-Wallis test) was used to compare continuous variables. Because only 32 patients had both eGFR < 60 ml/min/1.73 m^2^ and albuminuria, we examined the association between eGFR and albuminuria with anemia prevalence using chi-square test. For this, patients with eGFR< 60 ml/min/1.73 m^2^ were combined and compared with those eGFR ≥ 60 ml/min/1.73 m^2^. We also evaluated the associations of anemia with level of renal function (eGFR) using logistic regression. We repeated the analyses using mildly reduced eGFR (eGFR 60–89.9 ml/min/1.73 m^2^) in place of eGFR ≥ 90 ml/min/1.73 m^2^, as the reference group. Multivariate logistic regression analysis (backwards stepwise) was conducted and the corresponding adjusted odds ratios (AOR) and 95% confidence intervals (CI) were used to identify factors independently associated with anemia. *P* < 0.05 was used to indicate statistical significance.

### Ethical consideration

The study protocol was approved by the Institutional Review Board of College of Medicine and Health Sciences, Wollo University. Permission to conduct the study was also obtained from Dessie Referral Hospital. An informed verbal as well as written consent was obtained from each study participants. Physicians were informed about anemic and renal disease patients for proper management.

## Results

### Demographic and clinical characteristics of participants

Of the 422 participants, 412 had data on all the variables of interest and are included in the current cross sectional analysis. The demographic and clinical characteristics of the patients are shown in Table 1. The mean (± SD) age of patients was 45 ± 14.6 years; and 214 (55.7%) were females and 289 (70.1%) were type 2 diabetic patients. The median duration of diabetes was 4 years (25^th^–75^th^ percentiles, 2–6). Mean BMI was 22.23 ± 3.2 Kg/m^2^. Mean systolic and diastolic BP was 120 ± 19 and 79 ± 32 mmHg, respectively and 117 (28.4%) patients were hypertensive. The mean eGFR was 100.1 ± 39.8 ml/min/1.73 m^2^, and 59 patients (14.3%) had eGFR < 60 ml/min/1.73 m^2^. One hundred and fifty patients (36.4%) had albuminuria. On the other hand, about 79% of patients with albuminuria had eGFR ≥60 ml/min/1.73 m^2^. CKD was present in 43.0% of patients; of these, 35.0% had stage 1, 31.6% had stage 2, 32.2% had stage 3 and only one patient (0.6%) had stage 4 CKD (Table 1).

**Table 1:** Demographic and clinical characteristics of study participants (n = 412).

Characteristics		
Age (year), means ± SD		45 ± 14.6
Sex, n (%)	Male	183 (44.4)
	Female	229 (55.6)
Education, n (%)	< High school	243 (59.0)
	≥ High school	169 (41.0)
Type of diabetes, n (%)	Type 1	123 (29.9)
	Type 2	289 (70.1)
Duration of diabetes, n (%)	< 5 years	253 (61.4)
	5–10 years	107 (26.0)
	> 10 years	52 (12.6)
Antihypertensive medication, n (%)		113 (27.4)
Body mass index (Kg/m^2^), means ± SD		22.23 ± 3.19
Systolic BP (mmHg), means ± SD		120 ± 19
Diastolic BP (mmHg), means ± SD		79 ± 52
Hypertension, n (%)		117 (28.4%)
Fasting blood glucose (mg/dl), means ± SD		205.3 ± 69.7
Serum creatinine (mg/dl), means ± SD		1.01 ± 0.32
eGFR (ml/min/1.73 m^2^), means ± SD)		100.1 ± 39.8
Albuminuria, n (%)		150 (36.4)
Chronic Kidney Disease, n (%)		
Stage 1		62 (15.0)
Stage 2		56 (13.6)
Stage 3		58 (14.1)
Stage 4		1 (0.2)

### Prevalence of anemia

Of the 412 study patients, 110 (26.7%) had anemia according to the WHO criteria. Characteristics of patients with or without anemia are summarized in Table 2. Patients with anemia were significantly older (*P* < 0.001) and anemia prevalence progressively increased with age: 18.3% for age 18–30 years, 14.6% for 31–45 years, 31.4% for 46–60 years and 52.4% for >60 years (*P <* 0.001). Interestingly, males were more likely to be anemic than females (*P <* 0.001), but this may be related to the higher threshold of hemoglobin for males in the definition used. Patients with anemia were more likely to have type 2 diabetes, longer duration of diabetes, alcohol consumers, hypertension, high current systolic BP, fasting blood glucose, serum creatinine and low eGFR (all *P* < 0.05). There was no significant difference in the educational status, smoking, BMI and diastolic BP of patients with and without anemia.

**Table 2:** Characteristics of diabetic outpatients with and without anemia.

Variables	Anemia (n = 110)	No anemia (n = 302)	*P*–value*
Age (years)	51.7 ±15.8	42.7 ± 13.4	*P* < 0.001
Age (years), n (%)			*P* < 0.001
18–30	15 (18.3)	67 (81.7)	
31–45	19 (14.6)	111 (85.4)	
46–60	43 (31.4)	94 (68.6)	
>60	33 (52.4)	30 (47.6)	
Sex, n (%)			*P* < 0.001
Male	65 (35.5)	118 (64.5)	
Female	45 (19.7)	184 (80.3)	
Education, n (%)			*P* = 0.978
< High school	65 (26.7)	178 (73.3)	
≥ High school	45 (26.6)	124 (73.4)	
Type of diabetes, n (%)			*P* = 0.002
Type 1	20 (16.3)	103 (83.7)	
Type 2	90 (31.1)	199 (68.9)	
Duration of diabetes, n (%)			*P* < 0.001
≤ 10 years	74 (20.6)	286 (79.4)	
> 10 years	36 (69.2)	16 (30.8)	
Currently consume alcohol			*P* = 0.013
Yes	41 (35.3)	75 (64.7)	
No	69 (23.3)	227 (76.7)	
Current smoker			*P* = 0.752
Yes	14 (28.6)	35 (71.4)	
No	96 (26.4)	267 (73.6)	
Hypertension, n (%)			*P* < 0.001
Present	74 (65.5)	39 (34.5)	
Absent	36 (12.0)	265 (88.0)	
Body mass index (kg/m^2^)	22.6 ± 3.8	22.1 ± 2.9	*P* = 0.187
Systolic BP (mm Hg)	137.1 ± 21.4	113.9 ± 12.8	*P* < 0.001
Diastolic BP (mm Hg)	82.8 ± 11.1	77.7 ± 40.4	*P* = 0.379
Fasting blood glucose (mg/dl)	222.1 ± 73.5	199.2 ± 67.4	*P* = 0.003
Serum creatinine (mg/dl)	1.15 ± 0.40	0.96 ± 0.27	*P* < 0.001
eGFR (ml/min/1.73 m^2^)	91.4 ± 45.1	103.2 ± 37.4	*P* = 0.008

BP: blood pressure; eGFR: estimated glomerular filtration rate

*P-value calculated by chi-square test for categorical variables and student t-test for continuous variables

**Fig 1:**
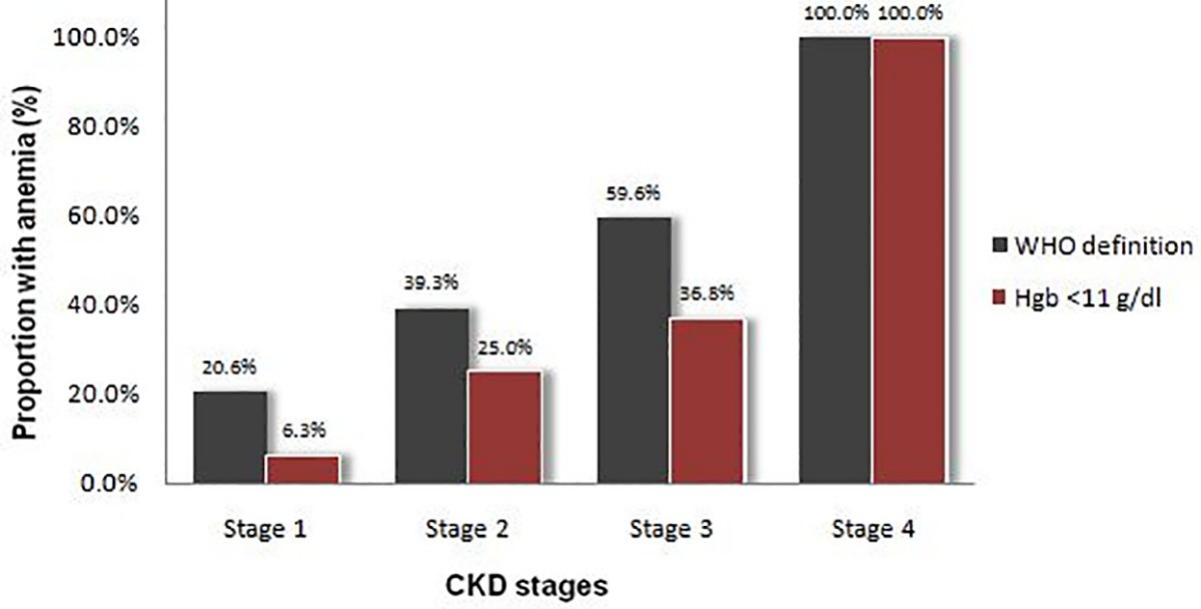
Distributions of anemia or haemoglobin <11 g/dl according to K/DOQI chronic kidney disease (CKD) stages.

Table 3 shows the prevalence of anemia in renal disease, namely CKD, impaired eGFR and albuminuria. Anemia was present in 39.5% of the patients with CKD compared to 17.0% of those without CKD (*P <* 0.001). The prevalence of anemia increased with stage of CKD, from 20.6% at stage 1 to 100.0% at stage 4 (Fig 1). Sixty-three of the total patients (15.3%) had anemia at levels where treatment is indicated, defined as a Hgb level < 11 g/dl, including 40 (22.6%) patients with CKD and 23 (9.8%) without CKD (*P* < 0.001). The prevalence of anemia at a Hgb level <11 g/dl increased significantly with worsening of CKD stages as expected.

**Table 3:** Prevalence of anemia in renal disease.

Renal disease	N (%)	Anemia, n (%)	*P*-value
Chronic kidney disease			*P* < 0.001
Present	177 (43.0)	70 (39.5)	
Absent	235 (57.0)	40 (17.0)	
eGFR (ml ⁄min/1.73 m^2^)			*P* < 0.001
< 60	59 (14.3)	35 (59.3)	
≥ 60	357 (85.7)	75 (21.2)	
Albuminuria			*P* < 0.001
Present	150 (36.4)	58 (38.7)	
Absent	262 (63.3)	52 (19.8)	

More than half (59.3%) of the patients with eGFR < 60 ml/min/1.73 m^2^ had WHO-defined anemia compared with 21.2% of those with eGFR ≥ 60 ml/min/1.73 m^2^ (*P* < 0.001). In addition, logistic regression revealed that patients with moderate renal impairment (eGFR = 30–59.9 ml/min/1.73 m^2^) were 6.3 times (OR = 6.32, 95% CI 3.41–11.73; *P* < 0.001) as likely to have anemia as patients with normal renal function (eGFR ≥ 90 ml/min/1.73 m^2^). Patients with moderate renal impairment were also 4.2 times (OR = 4.24, 95% CI 2.21–8.15; *P <* 0.001) as likely to have anemia as patients with mild renal impairment (eGFR = 60–89.9 ml/min/1.73 m^2^) (data not shown). When stratified by albuminuria status, 38.7% of those with albuminuria had anemia, about 2.5 times higher than the 19.8% in those without albuminuria (*P* < 0.001).

### Factors associated with anemia

Multivariate logistic regression analysis showed that old age >60 years (AOR = 2.41, 95% CI 1.11–5.21; *P =* 0.026); type 2 diabetes (AOR = 2.40, 95% CI 1.14–5.08; *P =* 0.022); presence of hypertension (AOR = 3.78, 95% CI 1.35–10.57; *P =* 0.011); current high systolic BP (AOR = 1.05, 95% CI 1.02–1.08; *P <* 0.001); high serum creatinine (AOR = 12.80, 95% CI 3.90–87.98; *P <* 0.001) and low eGFR (AOR = 9.50, 95% CI 4.05–22.28; *P <* 0.001) were independently associated with greater odds for the presence of anemia (Table 4).

**Table 4:** Factors associated with anemia among diabetic outpatients attending Dessie Referral Hospital, Northeast Ethiopia, 2018.

Variables	Crude OR (95%CI)	*P*–value	Adjusted OR (95%CI)	*P*–value
Age (years)				
≥ 60	1.00		1.00	
> 60	3.89 (2.23–6.77)	< 0.001	2.41 (1.11–5.21)	0.026
Sex			P < 0.001	
Male	2.25 (1.44–3.51)	< 0.001	1.12 (0.55–2.61)	0.653
Female	1.00		1.00	
Type of diabetes, n (%)				
Type 1	1.00		1.00	
Type 2	3.80 (1.88–7.67)	0.002	2.40 (1.14–5.08)	0.022
Duration of diabetes, n (%)				
≤ 10 years	1.00		1.00	
> 10 years	8.69 (4.57–16.52)	< 0.001	1.67 (0.68–4.08)	0.260
Currently consume alcohol				
Yes	1.80 (1.13–2.87)	0.013	1.23 (0.65–2.35)	0.523
No	1.00		1.00	
Hypertension, n (%)				
Present	13.86 (8.23–23.34)	< 0.001	3.78 (1.35–10.57)	0.011
Absent	1.00		1.00	
Systolic BP (mm Hg)	1.08 (1.06–1.10)	< 0.001	1.05 (1.02–1.08)	< 0.001
Fasting blood glucose (mg/dl)	(1.00–1.01)	0.004	1.01 (0.98–1.03)	0.895
Serum creatinine (mg/dl)	6.19 (3.03–12.65)	< 0.001	12.80 (3.90–87.98)	< 0.001
eGFR (ml/min/1.73 m^2^)				
≥ 90	1.00		1.00	
60–89.9	1.49 (0.89–2.50)	0.132	1.51 (0.82–3.09)	0.174
< 60	6.32 (3.41–11.73)	< 0.001	9.50 (4.05–22.28)	< 0.001
Albuminuria				
Present	2.55 (1.63–3.98)	< 0.001	1.23 (0.69–2.18)	0.502
Absent	1.00		1.00	

CI = confidence interval; eGFR: estimated glomerular filtration rate; OR = odds ratio

## Discussion

In this study, we found that the prevalence of anemia among diabetic adults attending our outpatient diabetes clinic of DRH was 26.7%. The overall prevalence of anemia observed in our study was higher than the 19% prevalence previously reported from the diabetes clinic of Fenote Selam Hospital, Northwest of Ethiopia [24]. The prevalence of anemia found in this study was; however, lower than the 41.4% prevalence reported in a tertiary care sub-Saharan African hospital study from Cameroon, which may be explained by the inclusion of only type 2 diabetic outpatients [25]. It was surprising to see that our prevalence estimate of anemia was comparable to 25% prevalence reported from the diabetes outpatients in Liverpool, UK which have better access to health services [26]. However, our prevalence estimate was higher than those reported in studies conducted elsewhere: including 14.6% in a tertiary center in Germany [27], 23.3% in the Austin and Repatriation Medical Center, Australia [10], 15% and 23.5% in the diabetes care clinics of Teesside and Liverpool, UK [28,29], but lower than the 41% prevalence reported from diabetic outpatients in London [14] and 55.5% in Saudi [30]. The difference might be due to variation in the geographical altitude [31], the age of the study population and the level of development of the country or due to the high prevalence CKD in our study subjects. The difference might also be due to variation in the study design, sampling techniques and sample size.

The current study also highlights a high prevalence of anemia, at levels where treatment is indicated, among diabetic patients whereby 15.3% of the patients had anemia below the recommended threshold for intervention (Hgb < 11 g/dl for both sexes). This prevalence of anemia is comparable to 14.3% prevalence reported from sub-Saharan African study in Cameroon [25]. Our prevalence estimate of anemia using the threshold recommended for intervention (Hgb < 11 g/dl) was higher than that of 5% reported in the Teesside, UK study [28], and 7% reported in the Australia study [10]. This finding underlines the need for regular hemoglobin testing to enable early identification of potentially treatable anemia among diabetic patients in primary care settings. In Ethiopia, anemia is not routinely screened at regular follow-up of diabetic patients except when patients present to hospital acutely with complications or other unrelated episodes and there is therefore a need to review our diabetes management.

Our findings of increased rates of anemia among diabetic patients with CKD compared to those without CKD is consistent with findings from previous studies evaluating the relationship of diabetes to anemia [13,14,32]. In this study 39.5% of diabetic patients with CKD were anemic, which was significantly higher than those without CKD (*P* < 0.001). This finding is significant given that anemia has previously been shown to be associated with cardiovascular disease and all-cause mortality and higher cost of managing diabetic patients with CKD [16,17]. The prevalence of anemia, according to WHO criteria, in our diabetic patients with CKD increased progressively with increasing CKD stages. This is in agreement with previous results [14,33–35], which suggested that anemia is prevalent at the earliest stages of CKD in diabetic patients and increases progressively with worsening of CKD stages. In the above study by Dimkovic N et al including both type 1 and type 2 diabetic patients, the presence of anemia increased progressively from 60% in stage 1 to 100% in stage 5 CKD [35]. The high prevalence of anemia, at levels where treatment is indicated, in our diabetic CKD patients, and its progressive increase with worsening of CKD stages is in accordance with previous studies, which suggests the importance of screening for anemia with renal disease in routine diabetes care [36].

Similar to previous reports, anemia in our study was more common among diabetic patients with impaired renal function (low eGFR) [10,26,24,25,37]. Fifty-nine percent of our participants with eGFR < 60 ml/min/1.73 m^2^ had anemia compared with 21.2% of those with eGFR ≥ 60 ml/min/1.73 m^2^. Renal function as measured by GFR equations was the strongest risk factor for prevalent anemia and may help to identify diabetic patients at greater risk [3]. Patients with diabetes and renal insufficiency have a higher risk of developing anemia associated with decreased production of erythropoietin by the failing kidneys [38]. It has been also suggested that patients with diabetes could constitute a significant additional burden to anemia in the presence of renal insufficiency. In this reared, a recent study advocated early screening, even at near-normal GFR, and more aggressive management of diabetic anemia with a view to improving quality of life and ultimate outcome for patients affected [14]. However, anemia was not confined to diabetic patients who had renal impairment; 19.7% of our patients had anemia with normal renal function (eGFR >90 ml/min/1.73 m^2^). This is in accordance with previous findings [10,19,25,37], which underlines the importance of assessing additional factors that may contribute to the increased risk for anemia in diabetic patients.

The multivariate analysis of this study showed that older age is independently associated with greater odds for the presence of anemia and this is consistent with other related studies [24,34,37]. The prevalence of anemia increase with age and it was two to threefold higher in those older than 60 years (52.4% vs. 22.1%), suggesting the need for routine screening for anemia in this high risk subgroup of patients. Anemia was also found to be highly prevalent among older patients with diabetes attending a long-term follow-up outpatient clinic, affecting 59% of patients [39]. The lack of association between duration of diabetes and anemia is not consistent with findings of a previous study in the country [24]. It seems that the duration of disease is not an important risk factor for anemia in our diabetic patients and what is important is the type of diabetes. Our analysis showed that the type of diabetes is an independent risk factor for anemia and this is consistent with other related studies [26,24]. This might be due to the fact that patients with type 2 diabetes pass through a period of pre-diabetes and may experience renal impairment at the time of diagnosis, thus exposing patients to high risk of anemia.

This study also showed a significant association between anemia and the presence of hypertension and current high systolic blood pressure. This association is of concern considering that hypertension in diabetic patients’ increases the risk of renal impairment and thus increasing the subsequent development of anemia. In the above study by Thomas et al, a weak association between systolic blood pressure and hemoglobin level was detected in diabetic patients with renal disease after adjusting for differences in renal function [10]. However, further studies are needed to evaluate the significant association of these factors with anemia in this population.

The present study has some limitations: the study lacks control groups and did not assess the causes of anemia in diabetic patients. Renal disease was assessed based on a one-time GFR and proteinuria measurements, possibly leading to overestimation of the actual prevalence of CKD. The cross-sectional nature of the study, where better relationship between anemia and different potential factors affecting it progressively cannot be well established, so a longitudinal study is needed to assess the relationship over time. However, the study has provided some data to inform decision-makers to improve current care and management of diabetic persons.

## Conclusions

In conclusion, anemia is commonly present among diabetic adults attending our outpatient diabetes clinic in Northeast Ethiopia, including those without kidney disease. More than a quarter (26.7%) of our diabetic outpatients had anemia and 15.3% had anemia below the recommended treatment threshold of 11 g ⁄ dl. Our findings highlight the need for incorporating anemia screening into the routine assessment of diabetic complications particularly for those with significant risk factors to enable early detection and treatment of anemia and hence improve the overall care of patients with diabetes.

## Supporting information

S1 File Data. (XLS)

## Abbreviations

AOR: Adjusted odds ratio
BMI: body mass index
CI: Confidence interval
CKD: Chronic kidney disease
DM: diabetes mellitus
DRH: Dessie Referral hospital
eGFR: Estimated glomerular filtration rate
FBG: Fasting blood glucose
Hgb: Hemoglobin
